# COVID-19 treatment strategies with drugs centrally procured by the German Federal Ministry of Health in a representative tertiary care hospital: a temporal analysis

**DOI:** 10.3205/id000083

**Published:** 2023-09-28

**Authors:** Kathrin Marx, Sven Kalbitz, Nils Kellner, Maike Fedders, Christoph Lübbert

**Affiliations:** 1 Hospital Pharmacy, St. Georg Hospital, Leipzig, Germany; 2 Department of Infectious Diseases and Tropical Medicine, St. Georg Hospital, Leipzig, Germany; 3 Division of Infectious Diseases and Tropical Medicine, Department of Medicine I, Leipzig University Hospital, Leipzig, Germany; 4 Interdisciplinary Center for Infectious Diseases, Leipzig University Hospital, Leipzig, Germany

**Keywords:** SARS-CoV-2, COVID-19, treatment, antiviral agents, monoclonal antibodies, variants of concern, pandemic waves

## Abstract

**Introduction:**

To ensure the fastest and earliest possible treatment, the German Federal Ministry of Health (BMG) initiated central procurement and nationwide distribution of new drugs against COVID-19. A single centre was used for a retrospective temporal analysis of this procedure.

**Methods:**

A descriptive analysis of hospitalization and treatment of COVID-19 patients with drugs centrally procured by the BMG at St. Georg Hospital, Leipzig, Germany, for the period from 1 March 2020 to 28 February 2023 was employed considering the approval status, evolving guidelines and recommendations of medical societies.

**Results:**

In total, 3,412 patients ≥18 years (54.9% men) with PCR-confirmed SARS-CoV-2 infection were admitted. The mean age was 64 years during the reporting period and 66.1/70.6 years during the first and second COVID-19 waves, respectively. 964 patients (28.2%) received COVID-19 therapy with drugs procured centrally by the BMG. Remdesivir was the most commonly used (63%). SARS-CoV-2 neutralizing monoclonal antibodies represented 23% of the therapies. Peroral antivirals (nirmatrelvir/ritonavir and molnupiravir) were used in 14% of COVID-19 patients, with molnupiravir being insignificant (five prescriptions).

**Conclusions:**

Specific therapeutic approaches were mainly based on antiviral therapy in the early phase of COVID-19 to prevent severe disease progression in vulnerable patient groups. Most drugs had not been approved at the time of central procurement; therefore, prescriptions were given on a case-by-case basis after careful risk–benefit assessments. All available neutralizing monoclonal SARS-CoV-2 antibodies lost efficacy during the pandemic due to different circulating immune escape variants. Remdesivir and nirmatrelvir/ritonavir remained effective therapies in the early phase of COVID-19.

## Introduction

Severe acute respiratory syndrome coronavirus type 2 (SARS-CoV-2) was first identified in December 2019 as the cause of a respiratory illness that developed in Wuhan (Hubei Province), China. The disease would thus be named coronavirus disease 2019 (COVID-19). Since the outbreak, the virus has spread worldwide rapidly, and the World Health Organization (WHO) declared COVID-19 a pandemic on 11 March 2020 [1]. By February 2023, 757 million COVID-19 cases and 6.85 million deaths had already been reported to the WHO worldwide [2]. On 27 January 2020, the first COVID-19 patient was diagnosed in Germany [3], and by 28 February 2023, more than 38 million people had contracted COVID-19, 894,000 people had been hospitalized and 167,951 people had died in Germany [4].

On 20 May 2020, the Federal Ministry of Health (BMG) issued an “Ordinance to ensure the supply of the population with products of medical need in the epidemic caused by the coronavirus SARS-CoV-2” (MedBVSV) to regulate the supply of urgently needed medicines and protective equipment as quickly as possible. Initially, 18 hospital pharmacies (star pharmacies) nationwide were designated as authorized agencies of the BMG in accordance with § 2 (1) and were charged with the storage, distribution and dispensation of the centrally procured medicines. According to § 3 (1), some regulations, including those of the German Medicines Act (AMG), did not apply to the appointed agencies. In particular, the provisions of § 43 (pharmacy obligation), § 47 (distribution channel), §§ 10, 11, 11a (labelling, package insert, expert information) and § 21 (authorization obligation) had to be applied [5]. To ensure the most uniform distribution of medicine possible, at the beginning of April 2021, the agencies commissioned by the BMG were expanded to include so-called ‘satellite pharmacies’ [6].

The centrally procured drugs included the direct antiviral agents remdesivir (Veklury), nirmatrelvir/ritonavir (Paxlovid), molnupiravir (Lagevrio) and the SARS-CoV-2 neutralizing monoclonal antibodies (mAbs) bamlanivimab/etesevimab, casirivimab/imdevimab (Ronapreve), sotrovimab (Xevudy) and tixagevimab/cilgavimab (Evusheld). The procurement and distribution channels for centrally procured drugs have adapted to therapy requirements in the course of the pandemic [7].

The Robert Koch Institute (RKI) established the Standing Working Group of Competence and Treatment Centers for Diseases Caused by Highly Pathogenic Pathogens (STAKOB) in 2014, which cooperates closely with the German Paul Ehrlich Institute (PEI) and the Federal Institute for Drugs and Medical Devices (BfArM). Following the current scientific findings of the SARS-CoV-2 pandemic, this nationwide network of experts published a statement on COVID-19 therapy and diagnostics for the first time on 8 April 2020. The 26^th^ update of the STAKOB statement was published on 8 February 2023 [8].

For a detailed understanding of the resulting treatment strategies in a representative hospital, a temporal investigation of the hospitalization and treatment of COVID-19 patients with the drugs centrally procured by the BMG was conducted at St. Georg Hospital, Leipzig. We focused on the temporal relationship between the provision and use of drugs in the context of the recommendations of the European Medicines Agency (EMA) and the resulting recommendations of professional societies. The aim of this analysis was to describe and classify the epidemiological development of hospitalization in the temporal context of the drugs procured centrally by the BMG, the recommendations of the EMA and specialist societies for targeted use, and the clinical implementation of the available therapy options.

## Methods

### Setting

St. Georg Hospital, Leipzig, Germany, is a tertiary care hospital with 1,066 plan beds and 25 different departments and clinics. Embedded in this structure is the Department of Infectious Diseases and Tropical Medicine as a STAKOB Centre for the Free State of Saxony. COVID-19 patients were primarily treated in the two infectious disease wards with a total of 44 plan beds and special isolation conditions. As a star pharmacy, the hospital pharmacy organized the storage, distribution, dispensation and patient-related documentation of the COVID-19 drugs procured centrally by the BMG.

### Study design and participants

In this retrospective study, only patients with polymerase chain reaction(PCR)-confirmed SARS-COV-2 infection were included. The study is limited to inpatients at St. Georg Hospital from 1 March 2020 to 28 February 2023. The following patient characteristics were recorded: age, sex, day of admission and discharge, length of stay, case mix index (CMI), discharge type and therapy with COVID-19 drugs centrally procured by the BMG.

### Epidemiological phases

The COVID-19 pandemic was classified into individual epidemiological phases and waves of disease in accordance with the phase classification of the RKI [9]:

- First wave (weeks 10–20, 2020), summer plateau 2020 (weeks 21–39)
- Second wave (week 40, 2020–week 8, 2021)
- Third wave (weeks 9–23, 2021), summer plateau 2021 (weeks 24–30)
- Fourth wave (weeks 31–51, 2021)
- Fifth wave (week 52, 2021–week 21, 2022)
- Sixth wave (week 22, 2022 onward)

During the pandemic, several SARS-CoV-2 variants were classified as variants of concern (VOCs). In particular, the proportion of dominant circulating VOCs was used to delineate the waves: Alpha for the third, Delta for the fourth, Omicron BA.1/BA.2 for the fifth and Omicron subline BA.5 for the sixth wave [9].

### Statistical analysis

Data analysis was based on descriptive statistics. The chi-square test was used for categorical variables, and the t-test was used for continuous variables to provide information on the totality of the data collection. Statistical analyses were performed using R statistical software (version 4.2.1).

## Results

### Patients

Within our three-year observation period, 3,412 patients suffering from COVID-19 with PCR-confirmed SARS-CoV-2 infection were hospitalized at St. Georg Hospital. Of these, 1,874 patients (54.9%) were men. The first COVID-19 patient was hospitalized at St. Georg Hospital on 6 March 2020. The respective absolute and relative number of patients between the first and sixth waves were as follows: first: 83 (2.4%), second: 627 (18.4%), third: 390 (11.4%), fourth: 433 (12.7%), fifth: 795 (23.3%), and sixth: 1,036 (30.4%). The clinical and demographic patient characteristics of the 3,412 cases according to the wave classification [9] are summarized in Table 1 (Tab. 1).

During the summer plateaus of 2020 and 2021, only individual cases were hospitalized. This finding aligns with a decline in the epidemiological infection rate. The average age of hospitalized patients was 64 years, with significantly higher ages during the first (66.1 years) and second (70.6 years) waves. In the second and sixth waves, the proportion of patients aged ≥80 years was 39.4% and 37.1%, respectively. The proportion of patients aged <18 years increased to 10.5% during the pandemic in the sixth wave.

### Application of drug therapy for COVID-19

**Figure 1:**
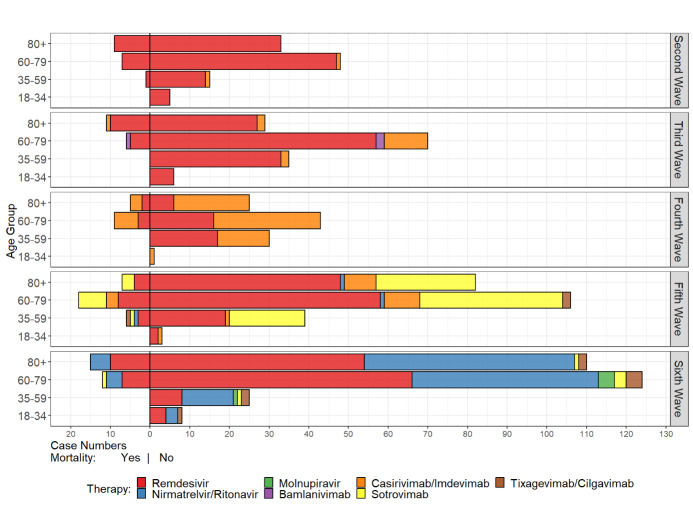
Summary of COVID-19 therapy by age group and mortality

A total of 964 (28.2%) adult patients (≥18 years) received therapy with COVID-19 drugs centrally procured by the BMG. The proportion was highest (40%) in the third wave. Twenty-four (12.4%) patients aged 18 to 34 years, 160 (26.9%) patients aged 35 to 59 years, 449 (36.7%) patients aged 60 to 79 years, and 331 (29.8%) patients aged ≥80 years received specific drug COVID-19 therapy. 107 (11.1%) patients died despite this specific therapy, of whom 100 (93.5%) were aged ≥60 years. Patients in the <18 years group did not receive COVID-19 drug therapy. Figure 1 (Fig. 1) summarizes the distribution of patients among age groups and COVID-19 drug therapy.

**Figure 2:**
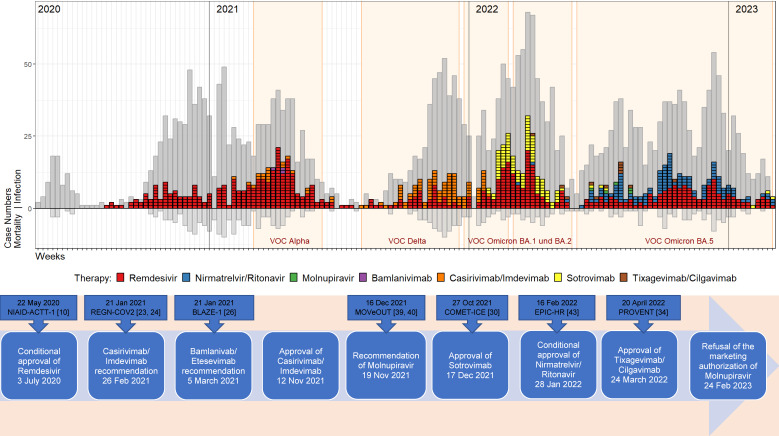
Temporal representation of the hospitalization and drug therapy of COVID-19 patients in the St. Georg Hospital, Leipzig, Germany, with special consideration of the drug therapy options provided by the BMG initiative

Remdesivir was the most commonly used drug, accounting for 63% of therapies. The SARS-CoV-2 neutralizing mAbs bamlanivimab/etesevimab, casirivimab/imdevimab, sotrovimab and tixagevimab/cilgavimab accounted for 23% of therapies. The peroral antiviral agents nirmatrelvir/ritonavir and molnupiravir were used in 14% of patients receiving therapy, with molnuparivir having no clinical significance (five prescriptions). Information on mortality, length of stay, and disease severity (CMI) was summarized for the clinical evaluation of antiviral therapies in Table 2 (Tab. 2). Only agents with meaningful case numbers (remdesivir, nirmatrelvir/ritonavir, casirivimab/imdevimab, and sotrovimab) were evaluated. The weekly course of infections and prescriptions at St. Georg Hospital is described in Figure 2 (Fig. 2).

## Discussion

The therapeutic approaches of the drugs centrally procured by the BMG were primarily based on antiviral therapy to avoid severe clinical courses. Nationwide, the direct antiviral agents remdesivir, nirmatrelvir/ritonavir, molnupiravir and the SARS-CoV-2 neutralizing mAbs bamlanivimab/etesevimab, casirivimab/imdevimab, sotrovimab and tixagevimab/cilgavimab were distributed. Most (71%) had not yet been approved for use in COVID-19 therapy during the initial centralized procurement. They were prescribed case-by-case for individual therapeutic trials and under careful benefit–risk consideration. Due to evolving study results, STAKOB’s evidence-based recommendations for action in the management of COVID-19 were regularly updated. The data were referenced to the main recommendations of the EMA and the time of the BMG’s central procurement of COVID-19 drugs.

Remdesivir (Veklury) was made available to St. Georg Hospital on 15 May 2020. This was followed by individual prescriptions as part of an individual cure trial. On 3 July 2020, remdesivir received conditional approval from the EMA based on the NIAID-ACTT-1 trial [10]. It was the first antiviral drug for COVID-19-affected adults and adolescents with pneumonia and supplemental oxygen requirements. With the COVID-19 guideline update on 20 November 2020, the WHO spoke out against the application of remdesivir [11] based on the interim results of the SOLIDARITY study [12] and a meta-analysis of data from all controlled trials. After the final evaluation and considering the WHO’s opinion, on 10 December 2020 the EMA implemented a restriction of the indication for remdesivir for patients with low-flow oxygen supplementation, high-flow oxygen supplementation or other non-invasive forms of ventilation [13]. Due to ongoing uncertainties regarding remdesivir’s benefits, the European Respiratory Society (ERS) and the Association of the Scientific Medical Societies in Germany (AWMF) did not advocate for or against remdesivir in their treatment guidelines [14], [15], [16]. Thus, uncertainties arose in prescribing remdesivir at St. Georg Hospital, resulting in its stagnant use in the second wave. By week 10 of 2021, case numbers and remdesivir therapy increased again, peaking in the third wave. During this wave 35.4% of patients (n=138) received remdesivir.

The third wave was particularly related to the emerging VOC Alpha, which had a higher replication rate [17]. Thus, the federal government extended federal lockdown measures, which resulted in consistently reduced infection rates across all states [18]. These lockdown measures and the vaccination of the particularly at-risk groups against SARS-CoV-2 have resulted in the containment of the rising infection rates in the third wave. As a result, this led to a reduction in the hospitalization rate of the most at-risk group. The average age of COVID-19 patients hospitalized at St. George Hospital decreased to 63 years. The proportion of those over 80 years of age decreased from 39% (second wave) to 23.6% (third wave). In December 2021, following the PINETREE study, the use of early therapy (within seven days of symptom onset) was ex-panded to patients with risk factors for a severe course [19]. Because remdesivir retained antiviral activ-ity in *in vitro* testing against the VOCs Alpha, Delta and Omicron with the BA.1/BA.2/BA.5 sublines [20], it was used at a rate of 63% throughout the pandemic. 69 (11.4%) patients died during remdesivir therapy. A recent meta-analysis on the effects of remdesivir in hospitalized COVID-19 patients summarized all-cause mortality at day 28 as the primary outcome of eight randomized controlled trials (RCTs) (10,480 patients) [21]. 12.5% of patients in the remdesivir group died by day 28, compared with 14.1% in the no remdesivir group (adjusted odds ratio [aOR] 0.88; 95% CI 0.78–1.00; p=0.045) [21]. In a separate retrospective analysis of SARS-CoV-2 patients admitted to St. Georg Hospital between 1 July 2021 and 30 June 2022, a significant reduction in 28-day mortality was confirmed in patients receiving remdesivir therapy [22].

The nationwide distribution of the SARS-CoV-2 neutralizing mAbs bamlavinimab and casirivimab/imdevimab began on 18 January 2021. The mAb combination casirivimab/imdevimab received EMA recommendations based on the REGN_COV-2 trial [23], [24] on 26 February 2021 [25]. The mAb combination bamlanivimab/etesevimab received an EMA recommendation based on the BLAZE-1 trial [26] on 5 March 2021 [27] for use in the EU. Both antibody therapies were indicated in adult COVID-19 patients who did not require oxygen and were not at increased risk for severe progression [28]. Notably, mAb bamlanivimab was used at St. Georg Hospital only in the third wave in three patients as a single-case decision. For the mAb combination bamlanivimab/etesevimab, the EMA halted the rolling review process on 2 November 2021 after the manufacturer (Eli Lilly) withdrew its marketing authorization application. The mAb combination casirivimab/imdevimab received European approval on 12 November 2021 as Ronapreve. With approval, casirivimab/imdevimab therapy also increased to 69 patients (60% of treated cases) in the fourth wave. The fifth wave was increasingly characterized by circulating VOC Omicron as the dominant variant, which caused a loss of efficacy of this mAb combination. It was only used at St. Georg Hospital at the beginning of the fifth wave, with 22 applications. A total of 111 patients received the mAb combination casirivimab/imdevimab, of whom 14 patients (12.6%) died. In the RECOVERY RCT, there was no significant difference in the primary endpoint of 28-day mortality between the casirivimab/imdevimab and standard treatment groups: 943 (19%) of 4,839 patients in the casirivimab/imdevimab groups died versus 1,029 (21%) of 4,946 patients in the standard treatment group (RR 0.94; 95% CI 0.86–1.02; p=0.14) [29]. The ninth week of 2022 saw the Omicron subline BA.2 become the predominant variant in Germany [9]. Subsequently, further use was no longer recommended in therapy or preexposure prophylaxis.

As of 17 December 2021, sotrovimab (Xevudy) was approved in the EU for adults and adolescents. Based on the COMET-ICE study [30], it was applied in cases not requiring oxygen supplementation but at increased risk for severe disease progression [30]. The first distribution of the contingents procured by the BMG to star pharmacies occurred on 18 January 2022. Sotrovimab was used in the fifth wave on 91 inpatients (35% of treated cases) at St. Georg Hospital. It demonstrated no change in antiviral activity in the Omicron subline BA.1 and a 15.7-fold reduction in BA.2. In the sixth wave (BA.5), sensitivity decreased by a factor of 21.6 [31]. At the beginning of the sixth wave, Sotrovimab was administered in double doses (1,000 mg) for only six patients at St. Georg Hospital [8]. A total of 97 patients received the mAb sotrovimab therapy, of whom 12 patients (12.4%) died. The mean CMI was significantly higher in deceased patients (4.57) than in convalescent patients (1.47). A significant difference was also found in length of stay: 38.5 days in deceased patients versus 13.7 days in convalescents. A systematic review and meta-analysis of 27,429 patients from 17 studies found a significant difference in mortality rates (OR=0.40, 95% CI 0.25–0.63; p<0.001) [32]. In a descriptive retrospective study of adult high-risk patients treated with sotrovimab, a mortality rate of 0.4% was observed. However, this study focused on outpatients, of whom 74 patients (2%) were hospitalized and of whom 9 patients (12.2%) died [33].

The central distribution of tixagevimab/cilgavimab (Evusheld) began on 18 February 2022. EMA approval was based on the PROVENT clinical trial [34] on 24 March 2022 [35]. Only 12 inpatients received preexposure prophylaxis at St. Georg Hospital. Based on the TACKLE study [36], an EMA approval extension [37] was granted on 15 September 2022. The extension included the treatment of COVID-19 disease for adults and adolescents who did not require supplemental oxygen and were at an increased risk for a severe course of COVID-19. This mAb combination was effective *in vitro* against Omicron sublines BA.1, BA.2 and BA.4/BA.5 with varying degrees of reduction in neutralizing capacity [38]. Currently, the efficacy of SARS-CoV-2 neutralizing monoclonal antibodies available in Germany is considered insufficient to justify monotherapy in the currently predominant sublineage of the Omicron variant [8].

The EMA published a recommendation on the use of molnupiravir (Lagevrio) on 19 November 2021 based on the MOVeOUT trial [39], [40]. It became available to all pharmacies nationwide on 3 January 2022 via distribution by pharmaceutical wholesalers (PHARGO). Molnupiravir was used at St. Georg Hospital in the sixth wave in only five patients as a single-case decision. Based on data available from February 2023, the EMA was unable to determine whether the use of molnupiravir can reduce the risk of hospitalization or death or shorten the time to recovery. With the rejection of the marketing authorization application, the basis for marketing under the MedBVSV ceased to apply on 24 February 2023 [41].

On 28 January 2022, the EMA granted conditional approval of the oral antiviral drug combination nirmatrelvir/ritonavir (Paxlovid) for the treatment of symptomatic, nonhospitalized COVID-19 patients without supplemental oxygen requirements and an increased risk of severe disease progression [42]. The approval was based on an interim analysis of the EPIC-HR study [43]. As of 25 February 2022, the drug was available nationwide via distribution by PHAGRO. Nirmatrelvir/ritonavir was the second most commonly used COVID-19 drug at St. Georg Hospital in the sixth wave, with 128 therapy units (42%). A total of 10 (7.8%) patients died during nirmatrelvir/ritonavir therapy. The mean CMI was significantly higher at 2.86 in deceased patients versus 1.40 in recovered patients (p=0.013). In a retrospective cohort study from Hong Kong, all-cause mortality was 3.6% in hospitalized patients with Omicron BA.2 infections receiving nirmatrelvir/ritonavir therapy [44]. The use of nirmatrelvir/ritonavir was associated with a significantly lower risk of death (HR 0.34, 95% CI 0.23–0.50; p<0.001) [44]. A systematic review with meta-analysis including 314,353 patients from 23 different studies found a significant difference in mortality as well (OR 0.25, 95% CI 0.14–0.45; p<0.001) [45]. Nirmatrelvir retained antiviral activity in *in vitro* assays against the variants of concern: Alpha, Delta and Omicron, with sublines BA.1/BA.2/BA.4/BA.5 [46]. Currently, nirmatrelvir/ritonavir is the drug of choice for adult COVID-19 patients who do not require supplemental oxygen and are at an increased risk of developing a severe course of COVID-19. Therapy should be initiated within the first five days of symptom onset or within five days of suspected infection [8].

Since the currently predominant SARS-CoV-2 Omicron variant sublines are associated with a lower risk of hospitalization and the proportion of severe illnesses and deaths is lower than in earlier waves, the RKI estimates the current overall risk from COVID-19 to be moderate [47]. Therefore, the BMG is not seeking further central procurement of COVID-19 drugs.

### Lessons learned

The possibility of rapid and early treatment of COVID-19 patients was successfully organized through central procurement of antiviral drugs and their nationwide distribution through the star and satellite pharmacies. However, without EMA approval, these drugs could only be prescribed as a case-by-case decision in the context of an individual treatment trial. EMA approval decisions were a prerequisite for the widespread clinical use of COVID-19 drugs.

All of the neutralizing SARS-CoV-2 monoclonal antibodies made available rapidly lost efficacy during the pandemic because of the different variants circulating. In clinical trials the direct antiviral drugs remdesivir (Veklury) and nirmatrelvir/ritonavir (Paxlovid) were shown to be effective therapeutic agents in the early phase of COVID-19 and were successfully incorporated into clinical treatment strategies. They retained antiviral activity against all eligible SARS-CoV-2 variants.

Under optimal treatment conditions, integrating a star pharmacy and a STAKOB center in a hospital, COVID-19 drug therapy was shown to have a positive impact on all-cause mortality in critically ill hospitalized patients.

## Notes

### Ethics approval

The study was conducted in accordance with the ethical guidelines of the 1964 Declaration of Helsinki and its later amendments and was approved by the local ethics committee (Saxonian Board of Physicians, Dresden, Germany, vote EK-BR-65/21–1).

### Authors’ contributions

K.M., M.F. and C.L. participated in the study conception and design. K.M. and S.K. were responsible for the acquisition of data from the study participants. K.M., S.K., N.K., M.F. and C.L. analyzed the data. K.M. performed the statistical analysis. K.M. and C.L. wrote the paper. All authors read and approved the final version to be published.

### Competing interests

The authors declare that they have no competing interests.

## Figures and Tables

**Table 1:**
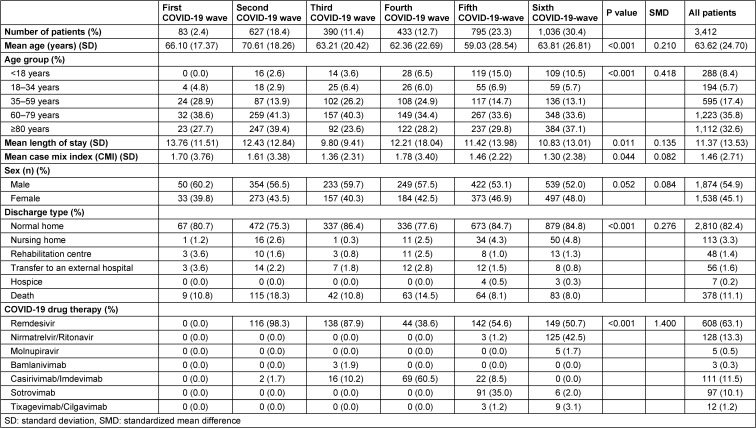
Patient characteristics of COVID-19 inpatients from 1 March 2020 to 28 February 2023

**Table 2:**
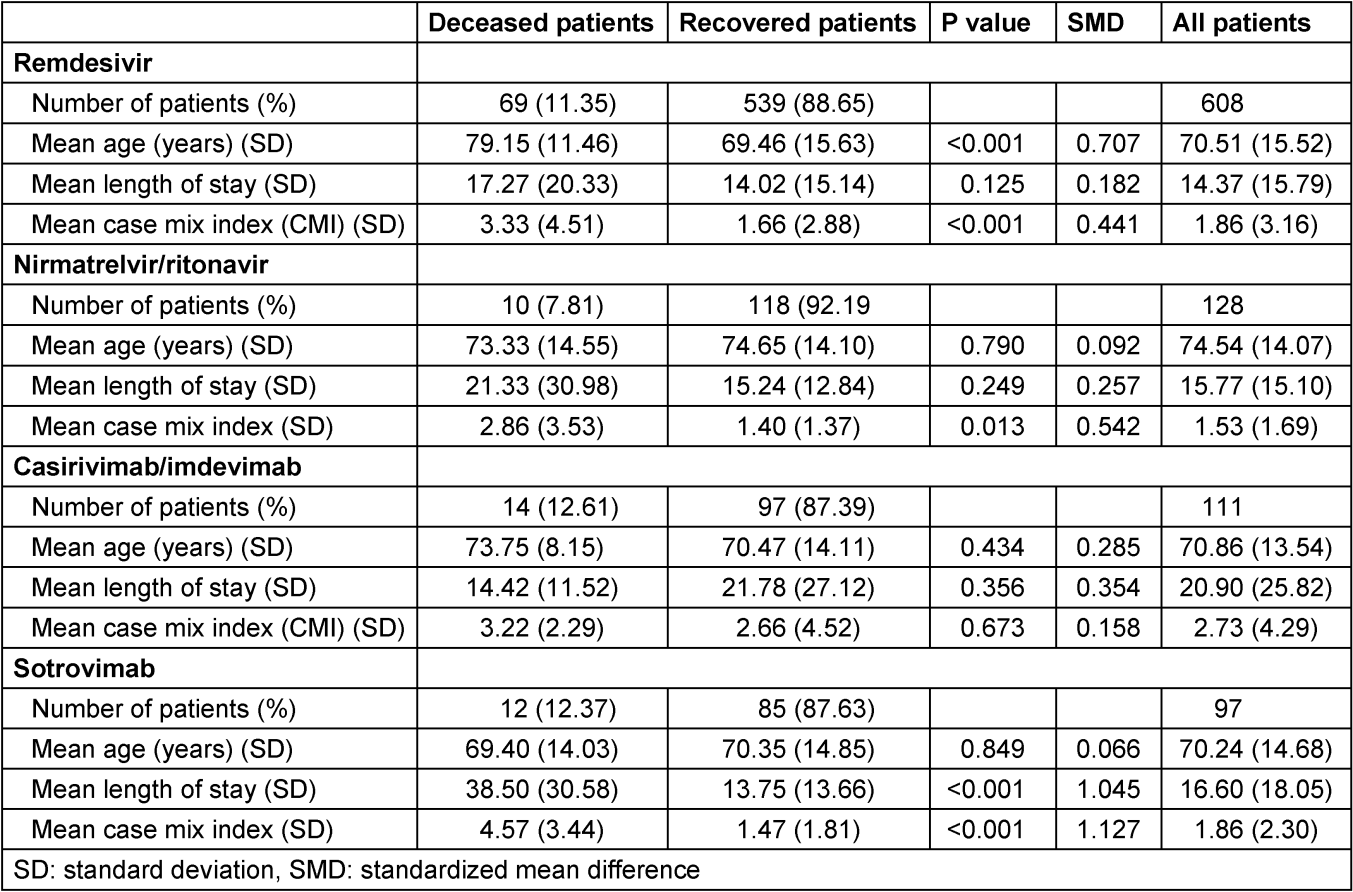
Clinical outcomes in patients receiving COVID-19 drug therapy
